# Optimal scan time for evaluation of parathyroid adenoma with [^18^F]-fluorocholine PET/CT

**DOI:** 10.1515/raon-2015-0016

**Published:** 2015-11-27

**Authors:** Sebastijan Rep, Luka Lezaic, Tomaz Kocjan, Marija Pfeifer, Mojca Jensterle Sever, Urban Simoncic, Petra Tomse, Marko Hocevar

**Affiliations:** 1 Department for Nuclear Medicine, University Medical Centre Ljubljana, Slovenia; 2 Department of Endocrinology, Diabetes and Metabolic Diseases, University Medical Centre Ljubljana, Slovenia; 3 Jozef Stefan Institute, Ljubljana, Slovenia; 4 Department of Oncological Surgery, Institute of Oncology Ljubljana, Ljubljana, Slovenia

**Keywords:** [^18^F]-fluorocholine PET/CT, lesions representing enlarged parathyroid tissue, triple-phase, standardized up-take value, retention index, lesion contrast

## Abstract

**Background:**

Parathyroid adenomas, the most common cause of primary hyperparathyroidism, are benign tumours which autonomously produce and secrete parathyroid hormone. [^18^F]-fluorocholine (FCH), PET marker of cellular proliferation, was recently demonstrated to accumulate in lesions representing enlarged parathyroid tissue; however, the optimal time to perform FCH PET/CT after FCH administration is not known. The aim of this study was to determine the optimal scan time of FCH PET/CT in patients with primary hyperparathyroidism.

**Patients and methods.:**

43 patients with primary hyperparathyroidism were enrolled in this study. A triple-phase PET/CT imaging was performed five minutes, one and two hours after the administration of FCH. Regions of interest (ROI) were placed in lesions representing enlarged parathyroid tissue and thyroid tissue. Standardized uptake value (SUV_mean_), retention index and lesion contrast for parathyroid and thyroid tissue were calculated.

**Results:**

Accumulation of FCH was higher in lesions representing enlarged parathyroid tissue in comparison to the thyroid tissue with significantly higher SUV_mean_ in the second and in the third phase (p < 0.0001). Average retention index decreased significantly between the first and the second phase and increased significantly between the second and the third phase in lesions representing enlarged parathyroid tissue and decreased significantly over all three phases in thyroid tissue (p< 0.0001). The lesion contrast of lesions representing enlarged parathyroid tissue and thyroid tissue was significantly better in the second and the third phase compared to the first phase (p < 0.05).

**Conclusions:**

According to the results the optimal scan time of FCH PET/CT for localization of lesions representing enlarged parathyroid tissue is one hour after administration of the FCH.

## Introduction

Primary hyperparathyroidism is an endocrine disorder that develops as a result of autonomous production and secretion of parathyroid hormone (PTH) from parathyroid glands. The most common cause of primary hyperparathyroidism is solitary adenoma (80–85%). Multi-glandular disease (multiple adenomas and parathyroid hyperplasia) is much rarer (15–20%).^1^ Parathyroid adenomas are benign monoclonal tumours that arise from neoplastic proliferation of a single abnormal cell. They are composed of main cells, oxyphil cells or a combination of both. In normally functioning parathyroid glands only a small number of cells are in the growth phase while in primary hyperparathyroidism the number of cells in the growth phase increases. The highest degree of proliferation is in parathyroid adenomas, followed by parathyroid gland hyperplasia.^2^,^3^

Symptomatic primary hyperparathyroidism is routinely treated with parathyroidectomy with a cure rate greater than 95% and a complication rate below 4%.^4^ Traditional surgical approach was a bilateral neck exploration with identification of all four parathyroid glands.^5^,^6^ Because most primary hyperparathyroidism cases can be attributed to a single adenoma^7^, a minimally invasive parathyroidectomy with selective exploration and excision of only abnormally functioning parathyroid glands can be performed. The first minimally invasive parathyroidectomy was performed in 1996.^8^ Since then, the minimally invasive parathyroidectomy became a mainstay treatment for single adenoma with primary hyperparathyroidism, providing decreased cost and patient discomfort^9^,^10^, and similar cure rates as a classic bilateral neck exploration.^11^ A prerequisite for successful minimally invasive parathyroidectomy is accurate preoperative localization of lesions representing enlarged parathyroid tissue (LREPT). [^99m^Tc]-sestaMIBI (MIBI) single-photon emission computed tomography/computed tomography (SPECT/CT)^12^ is a current gold standard for preoperative localization of LREPT with the sensitivity in the identification of a single adenoma of 80–90%.^13^–^15^ The ultrasonography that is often used in the preoperative or intraoperative setting as an adjunct for patients with negative MIBI scans, have the accuracy in identifying single adenomas of 70–80%.^16^,^17^ Both techniques have significantly lower accuracy for the detection of multi-glandular disease. Therefore, better imaging technique for preoperative localization of enlarged parathyroid glands is needed for wider acceptance of a minimally invasive parathyroidectomy.

[^11^C]-choline and [^18^F]-fluorocholine (FCH), the PET markers of cellular proliferation, was incidentally demonstrated to accumulate in LREPT.^18^ Therefore; FCH was proposed for the preoperative localization in patients with a primary hyperparathyroidism.^19^ In a pilot study, our group was the first to demonstrate the effectiveness of FCH in the preoperative localization of LREPT in patients with a primary hyperparathyroidism. In a group of 24 patients the performance of FCH PET/CT was superior to standard MIBI SPECT/CT particularly in patients with multiglandular disease.^20^

There are very limited data in the literature on the tissue kinetics of FCH and additionaly they are all from studies in prostate cancer patients. The time course of FCH accumulation and release from LREPT and adjacent thyroid tissue has not yet been described. The aim of present study was to determine the optimal scan time, i.e. time between radiopharmaceutical administration and FCH PET/CT imaging in patients with a primary hyperparathyroidism. At the optimal scan time the highest values of lesion contrast should be expected, but also the radiopharmaceutical properties, such as administered activity and isotope half-life as well as particular department logistics, especially radiopharmaceutical availability and scanner availability should be considered.

## Patients and methods

From May 2012 to May 2014, FCHPET/CT triple-phase point imaging was performed in addition to conventional MIBI SPECT/CT and neck ultrasound in 43 patients with a biochemically proven primary hyperparathyroidism (8 male and 35 female, mean age 59.6 ± 11 years; range 36–77 years). All patients had increased levels of preoperative calcium (mean 2.8 mmol/l; range 2.6–4.1 mmol/l; normal range 2.1–2.6 mmol/l) and increased preoperative iPTH levels (mean 311.5 ng/l; range 70.6– 2022 ng/l; normal range 10–65 ng/l). Patients with known history of malignant and/or inflammatory disease of the area of head and neck (other than autoimmune thyroid disease) were excluded from the study. All patients underwent surgery and had a histopathological examination of the removed parathyroid tissue. National medical ethics committee approved the study and informed consent was obtained from all patients.

After the administration of 100 MBq FCH (range: 96.8–104.5 MBq; mean 99.6 ± 2.2 MBq) the PET/CT imaging was performed at three time points: 5 minutes (first phase), one hour (second phase) and two hours (third phase). An integrated PET/CT scanner (Biograph mCT, Siemens) was used. At all three time points of the imaging process the neck and upper mediastinum were scanned in a single bed position with a scan time of 4 minutes, the time-of-flight information capture being enabled. The protocol included a low dose (120 kV; 25 mA) non-enhanced CT scan of the neck and upper mediastinum for the attenuation correction, followed by 3D PET acquisition in the same anatomical area. The images were reconstructed with iterative reconstruction with 2 iterations and 21 subsets, utilizing the scanner-specific point spread function. Data sets were reconstructed into standard 200×200×109 matrix size using a 4×4×2 mm^3^ voxel size. A 3D Gaussian post-reconstruction filtration with 4 mm full-width at half maximum was applied. All the images were acquired at the Department of Nuclear Medicine, University Medical Centre Ljubljana.

All FCH PET/CT images were masked and interpreted by two experienced observers on OASIS SEGAMI processing software. The image findings were scored for 5 different locations of LREPT: upper right/left, lower right/left and ectopic. Focally increased uptake outside the normal FCH biodistribution was estimate as positive for LREPT.

Scans of all three phases of FCH PET/CT were shown simultaneously on the monitor in the sagittal, transverse and coronary plane. Accumulated activity of FCH in LREPT was (semi)quantitatively valuated by placing a circular region of interest (ROI) adjusted to the metabolic volume of the gland. Maximum and mean standardized uptake values corrected for body weight (SUV_max_ and SUV_mean_) were obtained and SUV_mean_ was used for analysis. In the thyroid gland, the circular ROI was positioned in an area in the lateral lobe without any thyroid pathology on metabolic and anatomical (low dose CT) images. ROIs were copied and transferred to scans of all three time points, with automatic placement of the ROI in the appropriate location through automatic linking feature and manual correction if needed. Placement of ROIs was repeated four times to evaluate potential measurement error/dispersion.

For patients positive in all three phases, SUV_mean_ values were used to calculate the retention index (RI) and the lesion contrast (LC) – a quantitative measure obtained to evaluate the differential dynamics of tracer uptake in LREPT and thyroid tissue in order to determine optimal FCH PET/CT scan time. RI determines the percentage variation of the standard uptake values in LREPT and thyroid tissue and was compared between first-second and second-third phase.^26^ RI was calculated specifically for LREPT and thyroid gland. RI was calculated as:

[1] RIt,p=SUVt,pmeanlate−SUVt,pmeanearlySUVt,pmeanearly⋅100%

where the RI_t,p_ is retention index of LREPT (p) or thyroid tissue (t), and SUV_mean_ is standardized up-take values in LREPT (p) or thyroid tissue (t) for corresponding phase.^21^

LC refers to the difference in the visual intensity of LREPT and thyroid gland in the image that corresponds to different levels of radiopharmaceutical accumulation in these tissues. LC is important for the visual assessment of FCH PET/CT images and its higher value assists in the identification of abnormalities, because the radiotracer accumulation in LREPT is higher in most cases, but may also be lower in comparison to surrounding tissue; in such cases, the lesion contrast is negative. LC was calculated as:

[2] LC=SUVPmean−SUVTmeanSUVTmean⋅100%

where the LC is lesion contrast, ^P^SUV_mean_ is mean SUV in LREPT and ^T^SUV_mean_ is mean SUV in the thyroid tissue. LC was calculated for all three phases using the SUV_mean_ of LREPT and thyroid tissue for corresponding phase.^22^

FCH PET/CT results were compared with histopathological results, and sensitivity, specificity, positive predictive value (PPV), negative predictive value (NPV) and accuracy were calculated. SUV_mean_, RI and LC values are shown as average ± standard deviation (range). Group means were compared by two-tailed Student’s *t*-test for paired or unpaired data, as appropriate. A *p*-value < 0.05 was considered statistically significant. Statistical analysis was performed with the use of the SPSS software (version 16).

## Results

Sixty lesions of enlarged parathyroid tissue were localized by the FCH PET/CT scanning 43 patients. A primary hyperparathyroidism resolved and serum calcium normalized in 40/43 patients after surgery in which 60 parathyroid glands were removed (1.4 parathyroid gland/patient). According to histopathological diagnosis there were 34 solitary adenomas, one double adenoma, one cancer and hyperplasia in 7 patients (2/7 patients had a combination of primary and secondary hyperparathyroidism). Sensitivity, PPV, NPV and accuracy of the FCH PET/CT were higher in the second and third phase compared to the first phase (Table 1).

**FIGURE 1.**
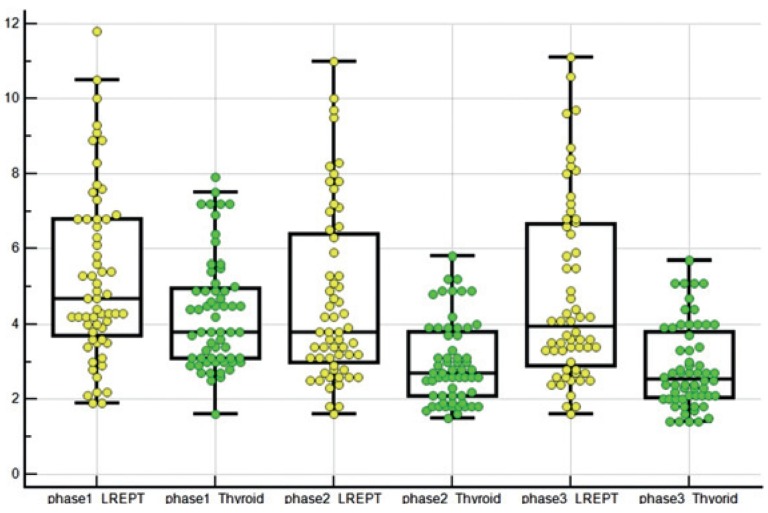
Different SUV_mean_ in all three phases of the described kinetics of FCH in LREPT and thyroid tissue.

**FIGURE 2.**
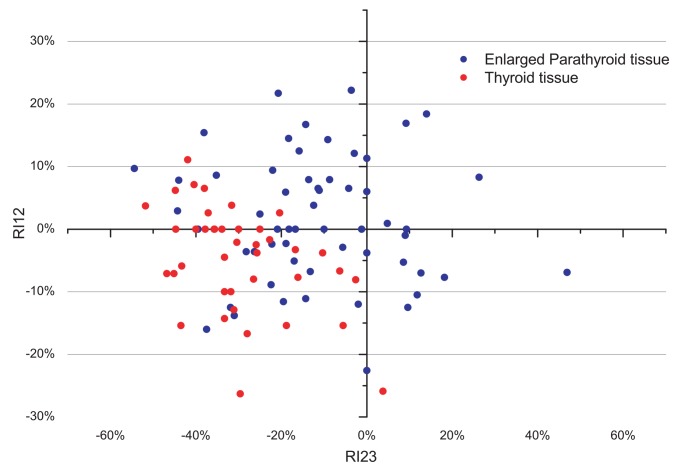
Retention index (RI) between the second and the third phase (RI23) versus RI between the first and the second phase (RI12) for all LREPT **(A)**, and for thyroid tissue **(B)**. Positive (negative) values of both RI12 and RI23 represent SUV_mean_ increase (decrease) through different phases; whereas positive (negative) RI12 and negative (positive) RI23 represent an increase (decrease) of SUV_mean_ after the first phase, followed by a decrease (increase) after the second phase.

Triple-phase PET/CT images showed a different distribution of FCH in the LREPT in comparison to the thyroid tissue (Figure 1, Table 2). On average, SUV_mean_ in LREPT was highest in the first phase, and then decreased significantly in the second phase (p < 0.0001), and increased non-significantly in the third phase (p = 0.2). Average SUV_mean_ in thyroid tissue was also highest in the first phase, then decreased significantly in the second (p < 0.0001) and the third phase (p = 0.009). The difference of the average SUV_mean_ between the LREPT and background thyroid tissues was significant in the second and the third phase. Figure 2 shows RI between the first and the second phase and between the second and the third phase. Average RI decreased significantly in LREPT between the first and the second phase, and increased significantly between the second and the third phase (both at p < 0.0001) (Table 3).

**FIGURE 3.**
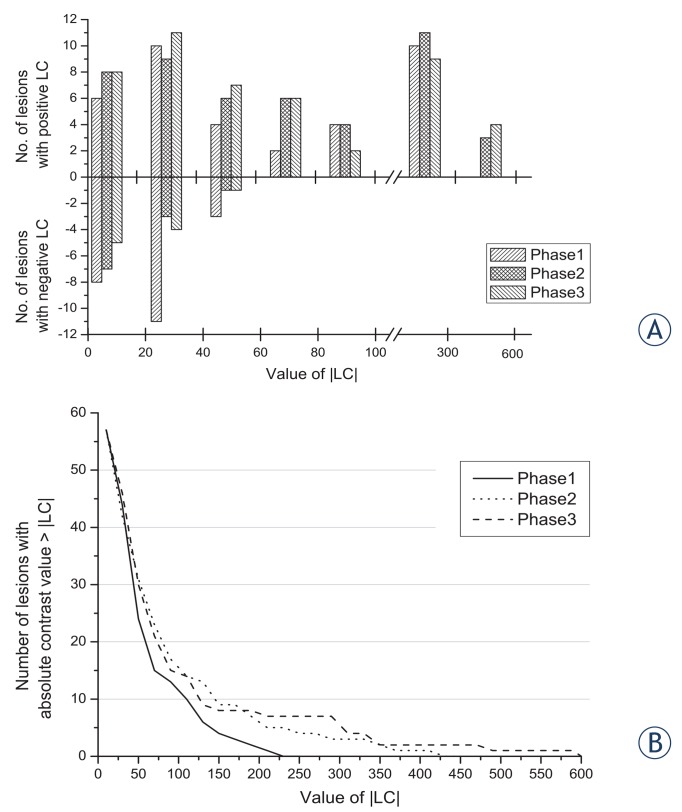
**(A)** The number of lesions in ranges of lesion contrast (LC) values for all three phases; for both positive and negative LC. **(B)** The number of both positive and negative lesions having absolute LC value equal or greater to the value on horizontal axis.

Observed LC of LREPT and thyroid tissue was 31.5% ± 60.8% in the first phase, 70.4% ± 95.5% in the second phase and 75.6% ± 121.5% in the third phase (Table 4).The difference in LC was statistically significant between the first and the second phase (p = 0.012) and between the first and the third phase (p = 0.015). The positive LC had values of up to 600% and the maximum negative LC had a value of 52%. Distribution of number of lesions along the ranges of LC values in all three phases is presented in Figure 3a. Additionally, in Figure 3b, the number of both – the positive and the negative - contrast lesions having the absolute LC greater than the selected value is presented.

## Discussion

MIBI SPECT/CT is the current gold standard for preoperative localization of LREPT in patients with a primary hyperparathyroidism with sensitivity in the identification of a single gland disease of 80–90%.^13^–^15^ However, in the case of multi-glandular disease the diagnostic performance of MIBI SPECT/CT is significantly lower.^23^ Therefore, better imaging techniques for preoperative localization of LREPT are being searched. Among different radiopharmaceuticals tested for PET scan in preoperative diagnosis of a primary hyperparathyroidism, [^11^C]-methionine is the most common one. However, the results of reported studies were not convincing enough to replace MIBI SPECT/CT.^24^–^26^ The first report of FCH accumulation in parathyroid adenomas and hyperplasia was based on incidental findings of Quak and Mapelli in patients with prostate cancer using FCH and [^11^C]-choline.^18^–^19^ It was our group that published the first study of comparison between FCH PET/CT and MIBI SPECT/CT and concluded that the FCH PET/CT is an accurate, efficient imaging modality for localization of hyper functioning parathyroid tissue, particularly in patients with multi glandular disease, where by its diagnostic performance is superior to the standard MIBI SPECT/CT.^20^ Better spatial resolution and LC are most probably responsible for higher sensitivity of FCH PET/CT.

**FIGURE 4.**
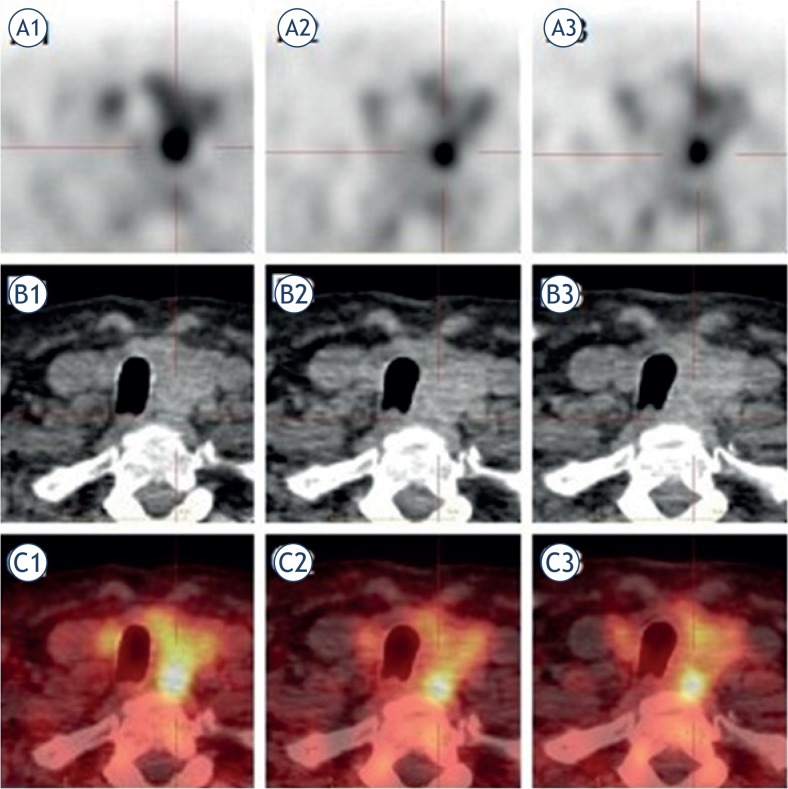
A secluded lower left LREPT. FCH PET/CT was performed in triple-phase after administration of 100 MBq of FCH. The LREPT is well delineated according to the thyroid tissue on PET axial image in the first **(A1)**, the second **(A2)** and the third phase **(A3)**. SUV_mean_ in the LREPT and the thyroid tissue was in the first phase 6.3 and 3.6, in the second phase 7.1 and 3, and in the third phase 6.6 and 2.9. The CT axial image of the LREPT and the thyroid tissue in all three phases **(B1, B2, B3)**, and the FCH PET/CT axial fusion image in all three phases **(C1, C2, C3)**.

The aim of our present study was to determine the optimal FCH uptake period that maximizes tumor-to-normal-tissue activity ratio. In order to determine the optimal scan time, SUV_mean_, RI and LC in LREPT and thyroid tissue were measured at three time points - 5 minutes (first phase), one hour (second phase) and two hours (third phase) after the administration of FCH (Figure 4).

Average accumulation of FCH was higher in LREPT in comparison to thyroid tissue in all three phases. However, the statistically significant difference in tracer uptake between LREPT and thyroid tissue, as assessed by SUV_mean_, was only found in the second and the third phase; in these two phases, LC was also significantly higher in comparison to the first phase. Additionally, in comparison to LREPT a higher SUV_mean_ in the thyroid tissue was found in more than a third (18/57; LREPT positive in all three phases) of lesions (8 solitary adenomas and 10 multi-glandular diseases). An underlying thyroid disease might be an explanation for a higher accumulation of FCH in the thyroid tissue. Three of these patients indeed had autoimmune thyroiditis; unfortunately, we did not have clinical data on thyroid disease status in the rest of these patients, but there was no known history of thyroid disease.

The highest SUV_mean_ value in LREPT was achieved shortly after the FCH administration and decreased gradually between the first and the second phase in the majority of lesions. Surprisingly, in approximately half of these lesions a slight increase of SUV_mean_ value in LREPT was observed between the second and the third phase. There is very limited data in the literature on the kinetics of FCH – all these studies include prostate cancer patients. Giussani *et al.*^27^ have developed a model of FCH kinetics based on biodistribution measurements that describes recirculation of radiopharmaceutical from major organs of early uptake (liver, spleen, kidneys) back into the blood pool, which may provide an explanation for late SUV increase in the parathyroid tissue. Tavola *et al.*^28^ concluded in their study that the simple linear model cannot adequately describe the kinetics of FCH, due to non-linear kinetics, which is associated with the release of FCH from the organs back into the blood. The non-linear kinetic model caused a slight overestimation of the activity in the liver and kidneys, most probably due to a physiological activity.

In addition to a higher accumulation of FCH in most LREPT in all three phases, in comparison to the thyroid tissue, there was also a slower (efflux) release of FCH from the LREPT, reflected by the highest LC between LREPT and thyroid tissue in the third phase. However, the difference in LC between the second and the third phase is not statistically significant, allowing PET/CT investigations to be performed from one to two hours after the administration of the radiopharmaceutical. Since the daily radiopharmaceutical dose was delivered to the department in the mornings, and its activity diminished relatively fast due to the 110 minutes half-life of 18F^29^, earlier scanning times were prefered. Therefore we are suggestting the optimal scan time of one hour with 4 minutes acquisition time. Such scanning protocol did allow us to image up to 12 patients with a primary hyperparathyroidism, each with small activities (100 MBq) administration of the radiopharmaceutical.

Despite the generally higher LC between LREPT and thyroid tissue in the second and the third phase, there was a single patient in our study with the uptake only in the first phase. In this patient’s case, an intense accumulation was present in the bone marrow in the second and the third phase, while almost no activity could be perceived in LREPT and thyroid tissue. A possible explanation for this unusual situation could be *polycythemia rubra vera*, which the patient was treated for. In order to avoid negative test results, early imaging might also be recommended in patients with potentially extensive hypermetabolic tissues, such as haematological and other malignancies.

Due to poor contrast between LREPT and thyroid gland in the first phase, three lesions were not localized, while in the second and the third phase the LC improved due to rapid wash out of FCH from the thyroid tissue and all three lesions were correctly localized. In one patient with hyperplasia two lesions were correctly localized as double adenomas, while two were false positive – histological results showed they were lymph nodes.

## Conclusions

Preoperative localization of parathyroid glands in patients with a primary hyperparathyroidism is possible with FCH PET/CT imaging. Optimal imaging time is one hour after the administration of FCH. Due to rare comorbidities, lesion uptake may be present exclusively in the early phase (immediately after administration) therefore if logistically possible, early phase imaging is recommended as well.

## Figures and Tables

**TABLE 1.** Comparison of binary classification test between all triple-phase FCH PET/CT

	**5 min FCH PET/CT**	**1 h FCH PET/CT**	**2 h FCH PET/CT**	**Together PET/CT**
Sensitivity	90.5%	93.6%	93.6%	95.3%
Specificity	98.2%	98.2%	98.2%	98.2%
PPV	96.6%	96.7%	96.7%	96.8%
NPV	94.7%	96.4%	96.4%	97.3%
Accuracy	94.1%	96.5%	96.5%	97.0%

**TABLE 2.** Comparison of average SUV_mean_ LREPT and thyroid tissue

**Tissue**	**Average SUV_mean_**		

	**5 min**	**1 h**	**2 h**
LREPT	5.29 ± 2.29 (1.9% to 11.8%)	4.69 ± 2.31 (1.6% to 11.0%)	4.77 ± 2.39 (1.6% to 11.5%)
Thyroid tissue	4.48 ± 1.55 (2.5% to 7.9%)	3.15 ± 1.11 (1.8% to 5.8%)	3.04 ± 1.13 (1.8% to 5.7%)
*p*	0.03	<0.0001	<0.0001

**TABLE 3.** Comparison of average RI in LREPT and thyroid tissue

**Tissue**	**Average RI**	

	**between first and second phase**	**between second and third phase**
LREPT	−11.1% ± 18.5% (−54.4% to 26.3%)	1,7% ± 10.2% (−22.8% to 22.2%)
Thyroid tissue	−29.8% ± 12.8% (−51.7% to 3.8%)	−4.5% ± 8.4% (−26.3% to 1.1%)
*p*	<0.0001	0.001

**TABLE 4.** Lesion contrast calculation and temporal comparison

	**5 min**	**1 h**	**2 h**
LC	31,1% ± 60.8% (−52% to 217.7%)	70.4% ± 95.5% (−44.8% to 410.5%)	75.6% ± 121.5% (−40.3% to 592.8%)

	**Between first and second phase**	**Between second and third phase**	**Between first and third phase**

Lesion contrast comparison *(p)*	0.012	0.8	0.015
